# Individual radiosensitivity reflected by γ-H2AX and 53BP1 foci predicts outcome in PSMA-targeted radioligand therapy

**DOI:** 10.1007/s00259-022-05974-8

**Published:** 2022-09-22

**Authors:** Liam Widjaja, Rudolf A. Werner, Elke Krischke, Hans Christiansen, Frank M. Bengel, Natalia Bogdanova, Thorsten Derlin

**Affiliations:** 1 Department of Nuclear Medicine, Hannover Medical School, Carl-Neuberg-Str. 1, 30625 Hannover, Germany; 2 Department of Radiation Oncology, Hannover Medical School, Carl-Neuberg-Str. 1, 30625 Hannover, Germany

**Keywords:** Prostate carcinoma, PSMA, Radioligand therapy, PSMA-PET, DNA damage response

## Abstract

**Purpose:**

γ-H2AX and 53BP1 are fundamental for cellular DNA damage response (DDR) after radiation exposure and are linked to cell repair, arrest, or apoptosis. We aimed to evaluate whether DDR-markers in peripheral blood lymphocytes (PBLs) may have predictive potential for outcome in metastatic castration-resistant prostate cancer (mCRPC) patients receiving [^177^Lu]Lu-prostate-specific membrane antigen (PSMA) radioligand therapy (RLT).

**Methods:**

We prospectively enrolled 20 men with advanced mCRPC scheduled for PSMA-targeted RLT. Prior to the first cycle of [^177^Lu]Lu-PSMA RLT, all patients underwent [^18^F]F-PSMA-1007 positron emission tomography (PET)/computed tomography (CT) for assessment of tumor PSMA expression (assessing maximum standardized uptake value (SUV_max_) of all tumor lesions). Blood samples were collected prior to, + 1 h after, and + 24 h after administration of [^177^Lu]Lu-PSMA, and DDR-markers γ-H2AX and 53BP1 were determined in PBLs through immunocytofluorescence. We then tested the predictive performance of DDR-markers relative to clinical and PET-based parameters for progressive disease (PSA-PD) after 2 cycles. In addition, the predictive value for progression-free survival (PSA-PFS, provided as median and 95% confidence interval [CI]) was explored.

**Results:**

Low baseline 53BP1 and γ-H2AX foci (*P* = 0.17) tended to predict early PSA-PD, whereas low SUV_max_ was significantly associated with higher risk for PSA-PD (*P* = 0.04). In Kaplan–Meier analysis, there was a trend towards prolonged PSA-PFS in patients with higher baseline 53BP1 of 6 months (mo; 95%CI, 4–9 mo) compared to 3 mo in patients with low 53BP1 (95% CI, 2–3 mo; *P* = 0.12). Comparable results were recorded for higher γ-H2AX expression (6 mo [95% CI, 3–9 mo] relative to 3 mo [95% CI, 2–4 mo] in patients with low γ-H2AX; *P* = 0.12). SUV_max_, however, did not demonstrate predictive value (*P* = 0.29). Consistently, in univariate Cox-regression analysis, baseline 53BP1 foci demonstrated borderline significance for predicting PSA-PFS under [^177^Lu]Lu-PSMA RLT (*P* = 0.05).

**Conclusion:**

In this prospective study investigating mCRPC patients undergoing [^177^Lu]Lu-PSMA RLT, low baseline DDR-markers in PBLs tended to predict poor outcome. Although the study group was small and results need further confirmation, these preliminary findings lay the foundation for exploring additive radiosensitizing or treatment intensification in future studies with high-risk individuals scheduled for RLT.

**Supplementary Information:**

The online version contains supplementary material available at 10.1007/s00259-022-05974-8.

## Introduction

Prostate-specific membrane antigen (PSMA)-targeted approaches have gained increasing acceptance for the diagnosis and treatment of patients afflicted with metastatic castration-resistant prostate cancer (mCRPC) [1–3]. As a type 2 transmembrane protein, PSMA is heavily overexpressed on the surface of prostate cancer cells [4], providing a target for radiolabeled imaging and therapy [1, 5–8]. For instance, [^177^Lu]Lu-PSMA-RLT demonstrated high efficacy and safety in a first prospective phase 2 study (LuPSMA) with over half of the investigated patients demonstrating a biochemical response [prostate-specific antigen (PSA) decline of over 50%] [1]. Further building on these promising results, Hofman et al. also recently revealed a favorable outcome for patients treated with [^177^Lu]Lu-PSMA-RLT when compared to the standard chemotherapeutic regimen using cabazitaxel [9]. Finally, the recently published prospective phase 3 VISION trial reported on longer progression-free (PFS) and overall survival for patients treated with [^177^Lu]Lu-PSMA-RLT compared to standard care [3, 10].

However, although patients had to demonstrate significant tumor PSMA expression to be enrolled into the VISION trial, 54% of the RLT-treated patients did not reach biochemical response [3]. In previous studies, high PSMA expression on pretherapeutic ^68^Ga-labeled PSMA-targeted PET/CT emerged as a strong predictor for treatment response [7, 11, 12]. Yet, all studies reported patients who did not respond although demonstrating increased PSMA expression [11–13]. In light of these results, more reliable predictors are needed. Gafita et al. recently reported on the predictive performance of nomograms containing both PET-based and clinical parameters [14], emphasizing the complexity of predicting treatment response in mCRPC patients receiving [^177^Lu]Lu-PSMA RLT. Thus, investigating the underlying pathophysiological response caused by RLT-based radiation might help to better understand the biological mechanisms of response and non-response.

Used for PSMA-targeted treatment, ^177^Lu as a primarily beta-emitting isotope induces a plurality of DNA damages, including DNA double-strand breaks (DSBs), ultimately leading to subsequent cell death [15, 16]. Following DSBs, a cascade of cellular DNA damage response (DDR) mechanisms is activated, involving the histone-variant γ-H2AX and the p53-binding protein 1 (53BP1) [16–18]. However, beta-radiation from [^177^Lu]Lu-PSMA does induce DSBs not only in tumor cells but also in benign cells such as peripheral blood lymphocytes (PBLs) which become exposed during circulation [19, 20]. The time course of DSB formation in PBLs and the potential to obtain data on the individual dose–response relationships in vivo have recently been reported in [^177^Lu]Lu-PSMA RLT [19, 20]. Marked interindividual heterogeneity on both amount and kinetics of DNA damage response (DDR) foci during [^177^Lu]Lu-PSMA RLT was also reported [19, 20]. Given the broad range of measured γ-H2AX and 53BP1 levels potentially reflecting individual radiosensitivity, these parameters may also hold potential for risk stratification, serving as “liquid biopsy” to identify patients that most likely benefit from RLT [21, 22].

In the present prospective study, we investigated the predictive performance of DSB markers relative to other established parameters, for the prediction of both early progression and progression-free survival in [^177^Lu]Lu-PSMA RLT.

## Materials and methods

### Patient population

Prior to their first treatment cycle, 20 mCRPC patients scheduled for [^177^Lu]Lu-PSMA-I&T RLT were included between August 2020 and Mai 2021 in this prospective monocentric study for assessing the value of DDR-markers in the context of therapy (Table 1). All patients were selected for [^177^Lu]Lu-PSMA-I&T RLT compliant to the German Medicinal Products Act, AMG §13.2b after progressing under previous treatments, including radiation therapy, androgen deprivation, and second-line antihormonal treatment, and had progressed under or were unfit for chemotherapy. In addition, all patients demonstrated PSMA-expression on pretherapeutic [^18^F]F-PSMA-1007 PET/CT. The institutional review board at Hannover Medical School approved this study (No. 9182_BO_S_2020), and written informed consent was obtained from all individuals. A detailed study protocol can be found in the Supplementary material.

**Table 1:** Patient characteristics (*n* = 20)

Variable	
Age (years, mean ± SD)	72.1 ± 8.6
Gleason score (mean ± SD)	9 ± 1
Previous treatments (%)	
Radical prostatectomy	45
Primary radiation therapy	15
Salvage radiation therapy	65
Androgen deprivation therapy	100
Enzalutamide	75
Abiraterone acetate	90
Previous chemotherapy	95
Docetaxel	95
Cabazitaxel	50
Laboratory values at baseline (mean ± SD)	
AST (U/I)	59 ± 84
ALT (U/I)	21 ± 12
AP (U/I)	241 ± 194
LDH (U/I)	515 ± 420
PSA (μg/L)	309.8 ± 418.3
Site of tumor lesions (%)	
Skeleton	100
Lymph nodes	90
Liver	10
Prostate/Prostate bed	55
PET-based parameters at baseline (mean ± SD)	
SUV_max_	19.4 ± 11.4
SUV_mean_	12.6 ± 8.2
PSMA-TV (cm^3^)	226.5 ± 195.8

*ALT*, alanine transaminase; *AP*, alkaline phosphatase; *AST*, aspartate transaminase; *LDH*, lactate dehydrogenase; *PET*, positron emission tomography; *PSA*, prostate-specific antigen; *PSMA*, prostate-specific membrane antigen; *RLT*, Radioligand therapy; *SUV*, standardized uptake value; *TV*, tumor volume

### Imaging procedure and interpretation

All PET/CTs were acquired on a dedicated PET/CT system (Biograph mCT 128 Flow; Siemens Healthineers; Knoxville, TN, USA), with an extended field-of-view for the PET and a 128-slice spiral CT. Per patient, 306 ± 23 MBq of [^18^F]F-PSMA-1007 was administered. Prior to PET acquisition, a low-dose non-enhanced helical CT (120 kV, mA modulated, pitch of 1.2, reconstructed axial slice thickness of 5.0 mm) for attenuation correction was performed. 100 min p.i. and after voiding of the bladder, whole-body PET was acquired using continuous bed motion at a speed of 0.9 mm/s for the chest and abdomen and 2.1 mm/s for the legs. All studies were reconstructed using Ultra HD (iterative algorithm with time-of-flight and point-spread function information including 2 iterations, 21 subsets; matrix, 200; zoom, 1.0; Gaussian filter, 5.0). No intravenous contrast material was injected.

For image analysis, a commercial software package enabling simultaneous and fused review of PET and CT data was used (syngo.via; V10B; Siemens Healthcare). Malignant lesions on all scans were segmented manually by one reader (L.W.) and verified by an experienced board-certificated specialist (R.A.W.), if necessary. To this end, an isocontour volume of interest was placed on individual lesions, including all voxels above 45% of the maximum using a 3-dimensional segmentation method allowing for a semi-automatic volumetric assessment [23]. This enabled for calculation of the whole-body PSMA-TV, averaged SUV_max_ (mean SUV_max_ of all tumor lesions in one patient), and averaged SUV_mean_ as described in [12].

### [^177^Lu]Lu-PSMA-I&T RLT

A GMP-compliant synthesis of [^177^Lu]Lu-PSMA-I&T was conducted [7, 24]. Per cycle, a median of 7.32 GBq (interquartile range [IQR], 7.28 to 7.37) of [^177^Lu]Lu-PSMA-I&T was administered. Treatment was performed following established procedure guidelines, with RLT cycles repeated every 6–8 weeks (median, 42 days [IQR, 42 to 43] [8]. Prior to the first and at each cycle, a standard laboratory panel, including blood cell counts, liver enzymes, and PSA was also assessed [25].

### Assessment of DDR-markers

As part of the study procedure, dedicated blood samples for the evaluation of DDR-markers were collected before administration of [^177^Lu]Lu-PSMA-I&T, + 1 h after administration and + 24 h after administration. Blood samples were prepared as previously described in [22]. In brief, after isolating PBLs through Ficoll (GE Healthcare, Chicago, IL, USA) density-gradient from EDTA blood, PBLs from mCRPC patients were frozen in an RPMI1640 medium with 15% fetal calf serum 500 U/ml penicillin, 0.5 mg/ml streptomycin, and 2 mM l-glutamine, supplemented with 10% glycerol at − 80 °C until further use. Subsequent PBLs were centrifuged on cover glasses (Cytospin ROTANTA 460/460R centrifuge, Hettich), fixed (3% (w/v) PFA, 2% (w/v) sucrose in PBS for 10 min and at RT) and permeabilized (0.2% (v/v) Triton X-100 in PBS for 3 min). Afterwards, cells were incubated simultaneously with antibodies against Phospho (S139)-Histone H2AX (Millipore, Burlington, MA, USA) in a ratio of 1:200 and against 53BP1 (Bethyl Laboratories, Montgomery, TX, USA) in a ratio of 1:400 for 1 h followed by at least three PBS washing steps and simultaneously incubation with Alexa Fluor anti-mouse IgG 488 and Alexa Fluor anti-rabbit IgG 546 (Invitrogen, Carlsbad, CA, USA; both at a ratio of 1:250) for another 45 min. Finally, DAPI (Invitrogen) was used to counterstain DNA and cells were mounted with ProLong® Gold (Invitrogen). Further details can be found in [22].

γ-H2AX and 53BP1 foci were counted under a Leica DMI6000B microscope (Wetzlar, Germany) using a 63 × objective lens at 1.6 × magnification with a manual focus through the whole nucleus enabling 3-dimensional foci detection. DDR-markers were assessed independently in several different areas of the slide leading to a median of 64 analyzed cells per sample. Cells demonstrating apoptotic morphology or an intensely stained nucleus were excluded from the analysis. In addition, monocytes and granulocytes were excluded from the analysis according to morphological criteria as described in [22]. DDR-markers related to intact PBLs were summed up and divided by the total number of PBLs to access the number of foci per cell.

### Assessment of clinical outcome

Before and 6–8 weeks after 2 cycles of RLT, PSA levels were determined and %-change to baseline was assessed. In patients discontinuing RLT, the last available PSA was used for response assessment. According to prostate cancer clinical trials working group 3 (PCWG3) criteria [26], treatment response after 2 cycles of RLT was determined. Early progressive disease (PSA-PD) was defined as a PSA increase of ≥ 25% from baseline, stable disease (PSA-SD) was defined as PSA-change between + 25% and − 50% and partial response (PSA-PR) as a PSA decrease over 50%. Patients without PSA-PD continued RLT with PSA measurements at and 4–6 weeks after each cycle. For the determination of progression-free survival (PSA-PFS), we calculated the time between the day of the first treatment cycle and the date of PSA-based PD as defined above. Patients were followed up until progression, termination of RLT due to other medical reasons, or the end of the follow-up period in December 2021.

### Statistical analysis

For statistical analyses, we used GraphPad Prism 9 (GraphPad Software, San Diego, CA, USA) and SPSS Statistics 27 Inc. (IBM; Chicago, IL, USA). We performed simple linear regression to demonstrate the association between the two DDR-markers γ-H2AX and 53BP1. Changes in DDR-marker levels at different time points were assessed through repeated-measures one-way ANOVA. Stratifying patients into high versus low values by median univariate logistic regression analysis was performed for DDR-marker levels and PET-derived and clinical parameters to explore the relationship of parameters with PSA-PD. The predictive performance for progression-free survival (PSA-PFS) of these parameters was then further assessed in Kaplan–Meier analysis and log-rank test. Finally, univariate Cox-regression for PSA-PFS was performed using continuous variables. A *P* value < 0.05 was considered significant.

## Results

### Inter- and intraindividual heterogeneity of DDR-markers during [^177^Lu]Lu-PSMA RLT

**Fig. 1:**
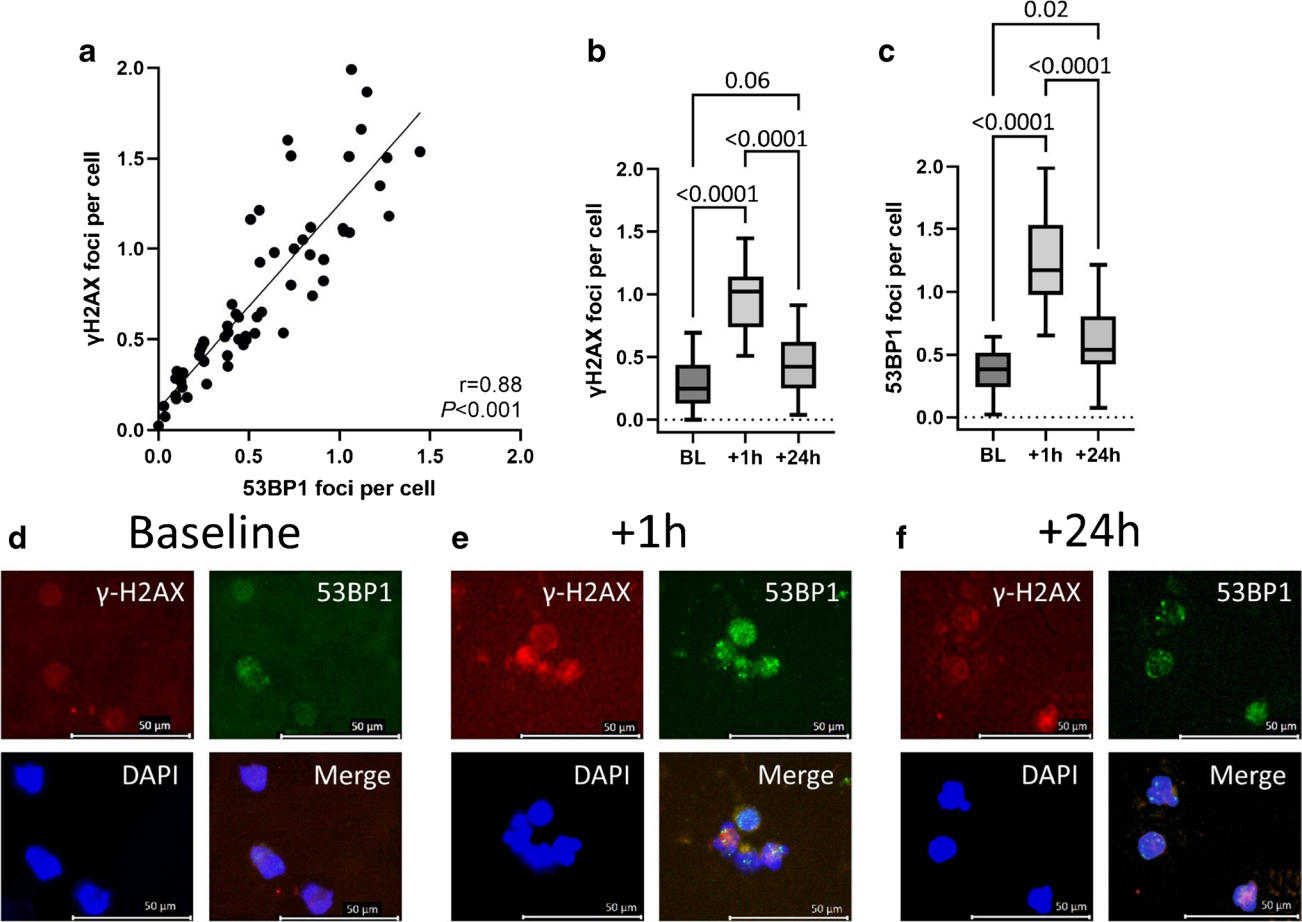
Evolution of DDR-markers during [^177^Lu]Lu-PSMA-RLT. There was a highly significant correlation between the DDR-markers γ-H2AX and 53BP1 (**a**). Both the number of γ-H2AX (**b**) and 53BP1 (**c**) foci increased significantly + 1 h after administration of [^177^Lu]Lu-PSMA and demonstrated a significant decrease after + 24 h. Compared to baseline, DDR-marker levels remained elevated at + 24 h after administration. Fluorescence microscopy images (63 × objective lens at 1.6 × magnification) of peripheral blood lymphocytes of a patient undergoing [^177^Lu]Lu-PSMA RLT, with one merged and three separate channels for γ-H2AX (*red*), 53BP1 (*green*), and DAPI for nuclei stain (*blue*). Compared to baseline levels (**d**), DDR-markers increased at + 1 h after administration of [^177^Lu]Lu-PSMA (**e**). Although there was a decrease at + 24 h (**f**), DDR-marker remained elevated when compared to baseline

A significant correlation between γ-H2AX and 53BP1 foci was observed (*r* = 0.88, *P* < 0.001; Fig. 1a), thereby validating the herein-established model for measuring DDR-markers in PBLs. In addition, we observed marked interindividual heterogeneity of DDR-marker levels at baseline, + 1 h and + 24 h (Table 2). Both γ-H2AX and 53BP1 foci per cell increased significantly from baseline to + 1 h, followed by a subsequent decrease at + 24 h (*P* < 0.0001, respectively). In addition, 53BP1 and γ-H2AX foci remained elevated relative to baseline at + 24 h (53BP1, *P* = 0.02; γ-H2AX, *P* = 0.06; Fig. 1b-f).

**Table 2:** Evolution of DDR-markers during [^177^Lu]Lu-PSMA RLT

Variable		*P* value*
Baseline		
γ-H2AX foci per cell	0.29 ± 0.19	
53BP1 foci per cell	0.37 ± 0.17	
+ 1 h after administration		
γ-H2AX foci per cell	0.95 ± 0.26	< 0.0﻿001
53BP1 foci per cell	1.27 ± 0.36	< 0.0001
+ 24 h after administration		
γ-H2AX foci per cell	0.45 ± 0.26	0.06
53BP1 foci per cell	0.61 ± 0.31	0.02

*RLT*, radioligand therapy. *P value of repeated-measures one-way ANOVA compared to baseline values

### Prediction of early treatment response

4/20 (20%) RLT-naïve patients achieved PSA-PR, whereas 9/20 (45%) had PSA-SD and the remaining 7/20 (35%) PSA-PD.

Stratifying patients into low versus high values by median, only 20% of the patients with higher baseline 53BP1 or γ-H2AX foci per cell demonstrated PSA-PD, whereas in patients with lower baseline DDR-markers, 50% suffered from early PSA-PD. In line with these results, there was a trend for both higher baseline 53BP1 foci per cell (Odds Ratio [OR], 0.25, 95% confidence interval [CI], 0.034–1.819, *P* = 0.17) and higher baseline γ-H2AX foci per cell (OR, 0.25, 95%CI, 0.034–1.819, *P* = 0.17) reducing the likelihood of PSA-PD in univariate logistic regression (Table 3). In addition, higher SUV_max_ (OR, 0.074, 95%CI, 0.007–0.835, *P* = 0.04), but not PSMA-TV (*P* = 0.64), significantly reduced the risk for PSA-PD, as well. Among clinical parameters, elevated AP (OR, 13.5, 95%CI, 1.197–152.211, *P* = 0.04) was predictive for PSA-PD.

**Table 3:** Univariate predictors of early PSA-progression

Variable	Odds ratio	95% CI	*P* value
Clinical parameters at baseline			
AST (U/l)	4	0.55–29.096	0.17
ALT (U/I)	4	0.55–29.096	0.17
AP (U/I)	13.5	1.197–152.211	0.04*
LDH (U/I)	4	0.55–29.096	0.17
PSA (μg/l)	0.25	0.034–1.819	0.17
Baseline PET			
SUV_max_	0.074	0.007–0.835	0.04*
PSMA-TV (cm^3^)	0.643	0.101–4.097	0.64
DDR-markers			
Baseline			
γ-H2AX foci per cell	0.25	0.034–1.819	0.17
53BP1 foci per cell	0.25	0.034–1.819	0.17
+ 1 h after administration			
γ-H2AX foci per cell	1.556	0.224–9.913	0.64
53BP1 foci per cell	4	0.55–29.096	0.17
+ 24 h after administration			
γ-H2AX foci per cell	1.556	0.224–9.913	0.64
53BP1 foci per cell	1.556	0.224–9.913	0.64

*ALT*, alanine transaminase; *AP*, alkaline phosphatase; *AST*, aspartate transaminase; *CI*, confidence interval; *LDH*, lactate dehydrogenase; *PSA*, prostate-specific antigen; *PSMA*, prostate-specific membrane antigen; *TV*, tumor volume; *SUV*, standardized uptake value; *DDR*, DNA damage response. *Reached significance

### Prediction of PSA-PFS

**Fig. 2:**
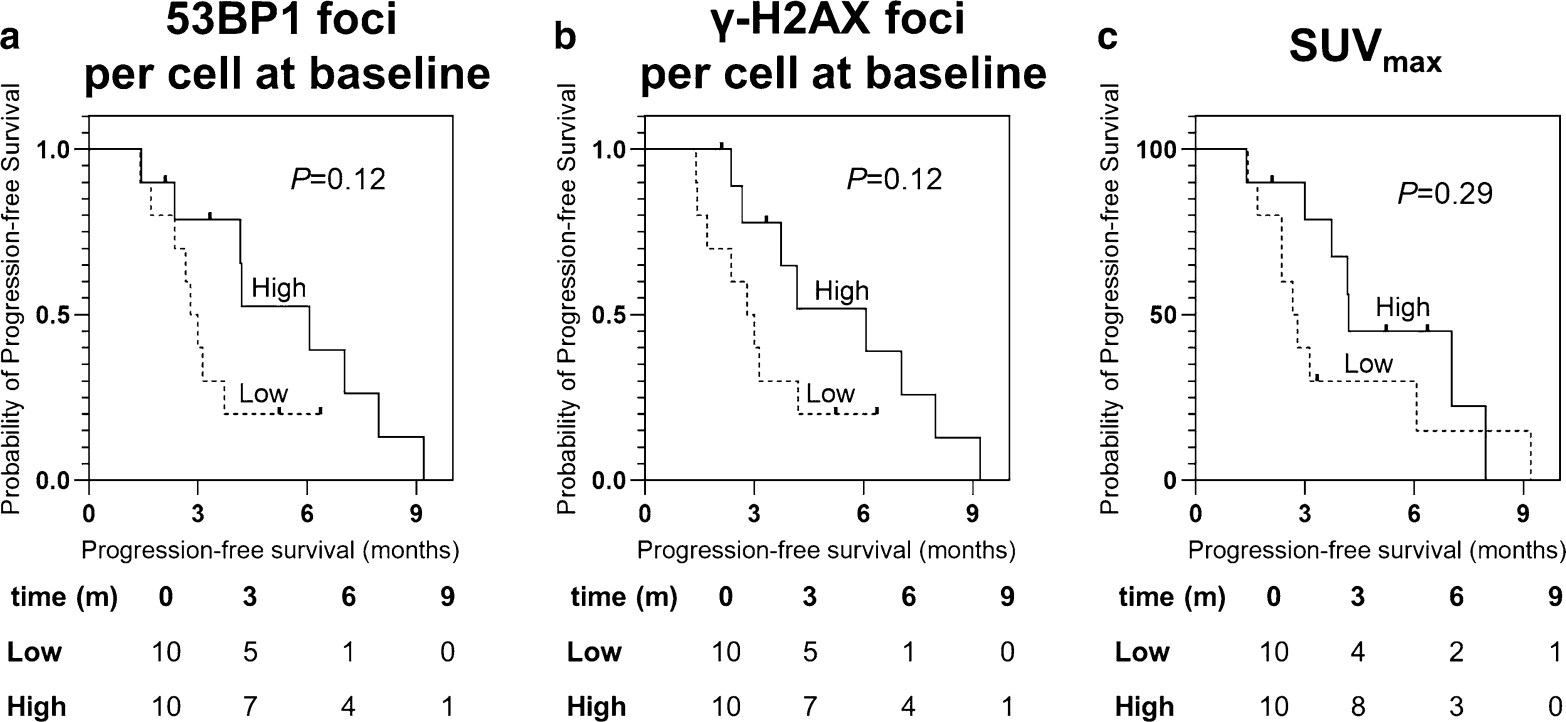
Kaplan–Meier analysis for PSA-progression-free survival (PFS). There was a clear trend for high baseline 53BP1 foci (**a**) and high baseline γ-H2AX foci (**b**) predicting prolonged PFS. SUV_max_ derived from pretherapeutic [.^18^F]F-PSMA-1007 PET did not reach significance for PFS (**c**)

In the Kaplan-Maier analysis (Fig. 2), there was a trend towards prolonged median PSA-PFS in patients with higher baseline 53BP1 (6 months [mo; 95% CI, 4–9 mo] compared to 3 mo [95% CI, 2–3 mo] in patients with low 53BP1; *P* = 0.12*)* and higher γ-H2AX expression (6 mo [95% CI, 3–9 mo] compared to 3 mo [95% CI, 2–4 mo] in patients with low γ-H2AX; *P* = 0.12*)*, albeit not statistically significant. In contrast, patients with high SUV_max_ only achieved a median PSA-PFS of 4 mo (95% CI, 4–4 mo) compared to 3 mo (95% CI, 2–3 mo) in patients with low SUV_max_ (*P* = 0.29). In line with these results, baseline 53BP1 foci per cell demonstrated borderline significance (Hazard Ratio [HR], 0.24 (95%CI, 0.001–1.025), *P* = 0.05) in univariate Cox-regression for PSA-PFS (Table 4). In addition, baseline γ-H2AX foci per cell (*P* = 0.1) and SUV_max_ (*P* = 0.06) showed borderline significant associations. Among clinical parameters, AP (*P* = 0.03) was predictive for PSA-PFS.

**Table 4:** Results of univariate Cox-regression analysis for PSA-progression-free survival

Variable	Hazard ratio	95% CI	*P* value
Clinical parameters at baseline			
AST (U/l)	1	0.993–1.007	0.98
ALT (U/I)	1	0.957–1.044	0.99
AP (U/I)	1.003	1–1.005	0.03*
LDH (U/I)	1.001	1–1.002	0.2
PSA (μg/l)	1	0.998–1.001	0.59
Baseline PET			
SUV_max_	0.926	0.855–1.003	0.06
PSMA-TV (cm^3^)	0.99	0.997–1.002	0.66
DDR-markers			
Baseline			
γ-H2AX foci per cell	0.054	0.002–1.819	0.1
53BP1 foci per cell	0.024	0.001–1.025	0.05
+ 1 h after administration			
γ-H2AX foci per cell	1.779	0.164–19.336	0.64
53BP1 foci per cell	1.897	0.421–8.539	0.4
+ 24 h after administration			
γ-H2AX foci per cell	1.565	0.106–23.167	0.74
53BP1 foci per cell	1.227	0.21–7.176	0.82

*ALT*, alanine transaminase; *AP*, alkaline phosphatase; *AST*, aspartate transaminase; *CI*, confidence interval; *LDH*, lactate dehydrogenase; *PSA*, prostate-specific antigen; *PSMA*, prostate-specific membrane antigen; *TV*, tumor volume; *SUV*, standardized uptake value; *DDR*, DNA damage response. *Reached significance

**Fig. 3:**
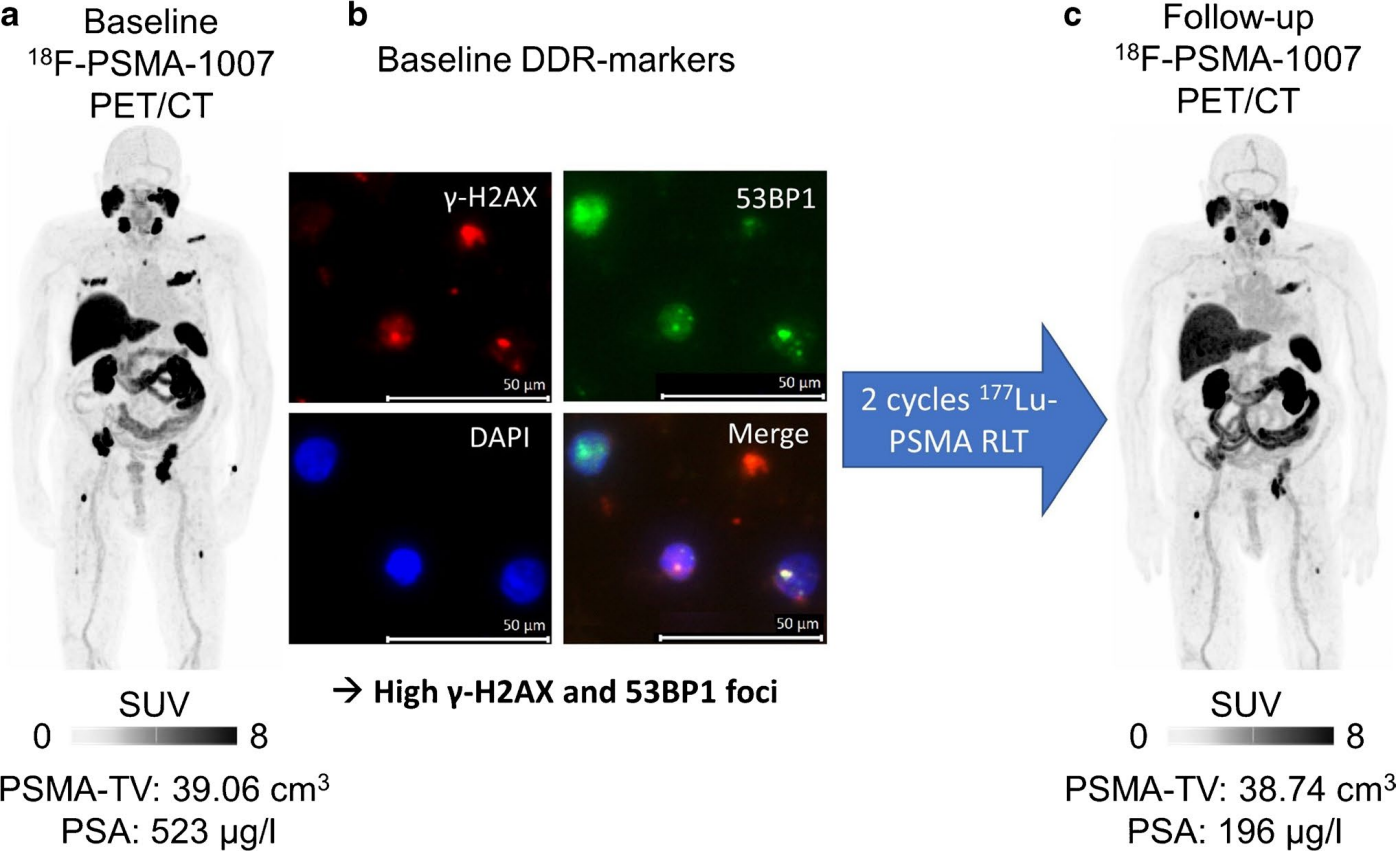
High DDR-marker levels are associated with treatment response. Baseline [^18^F]F-PSMA-1007 PET/CT (maximum intensity projection (MIP, **a**) of a patient scheduled for RLT. Multiple PSMA-positive lesions in the skeleton can be seen, e.g., in the ribs or in the left acetabulum. Averaged SUV_max_ was 14.6 (below median, indicative of progressive disease). Pretherapeutic PSA was 523 µg/l. Fluorescence microscopy images (63 × objective lens at 1.6 × magnification), with one merged and three separate channels for γ-H2AX (*red*), 53BP1 (*green*), and DAPI for nuclei stain (*blue*) of DDR-markers in PBLs prior to treatment (**b**). This patient demonstrated a rather high number of γ-H2AX (*n* = 0.44) and 53BP1 (*n* = 0.5) foci per cell, presumably reflecting high radiosensitivity. This patient achieved partial response after 2 cycles of [.^177^Lu]Lu-PSMA RLT (**c**; PET: 66 days after study inclusion; PSA: 84 days after study inclusion, − 63% compared to baseline)

**Fig. 4:**
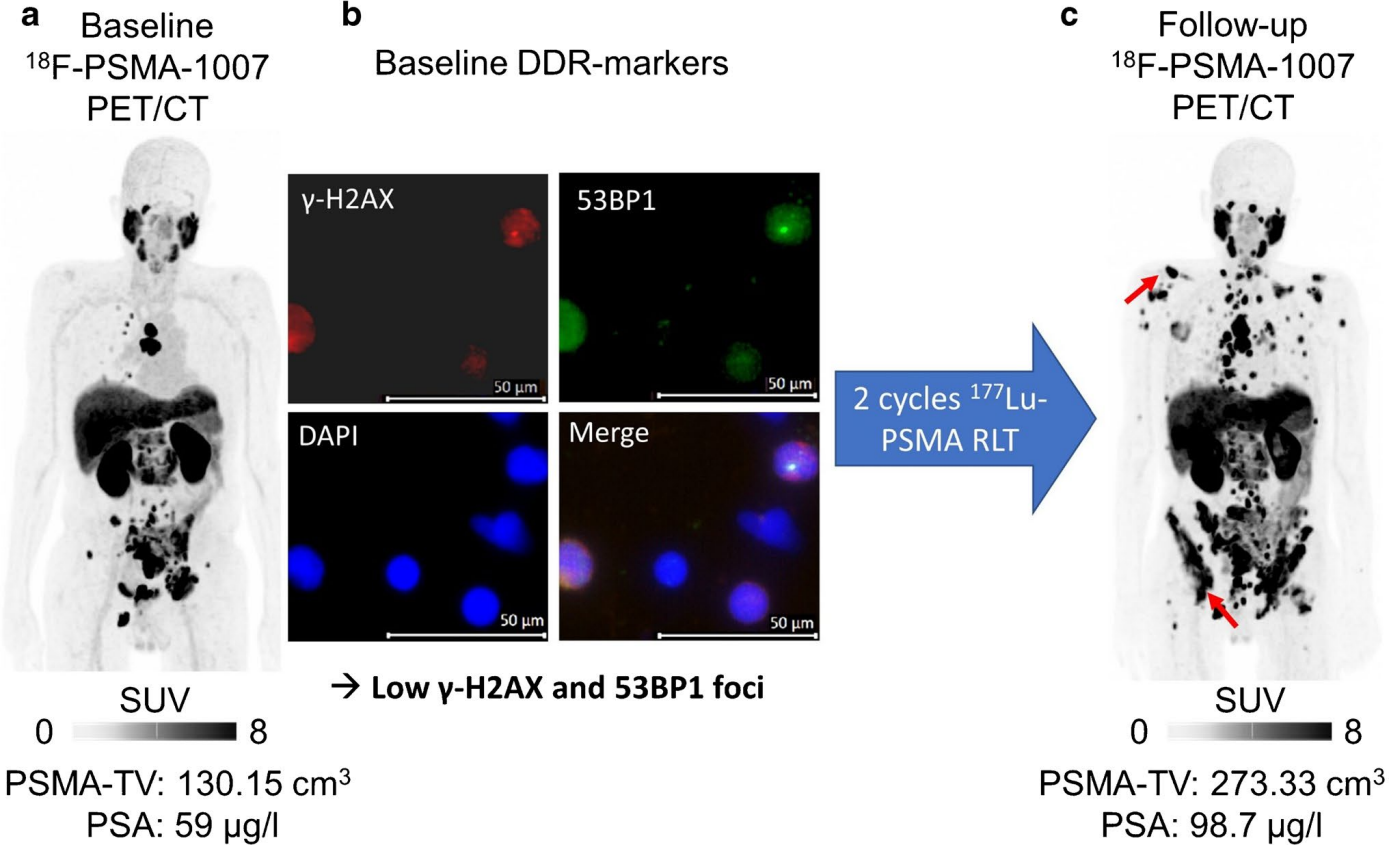
Low DDR-marker levels are associated with treatment failure. Baseline [^18^F]F-PSMA-1007 PET/CT (maximum intensity projection (MIP), **a**) of a patient prior to treatment. This patient had multiple tumor lesions in lymph nodes and the skeleton, e.g., in the11^th^ thoracic vertebral body. Averaged SUV_max_ was 18.39 (above median, indicative of no progressive disease). Pretherapeutic PSA was 59 µg/l. Fluorescence microscopy images (63 × objective lens at 1.6 × magnification), with one merged and three separate channels for γ-H2AX (*red*), 53BP1 (*green*), and DAPI for nuclei stain (*blue*) of DDR-markers in PBLs prior to treatment (**b**). This patient demonstrated a low number of γ-H2AX (*n* = 0.16) and 53BP1 (*n* = 0.18) foci per cell, presumably reflecting low radiosensitivity. This patient had progressive disease after 2 cycles of [^177^Lu]Lu-PSMA RLT (**c**; PET: 71 days after study inclusion, multiple new lesions (*red arrows*) on follow-up PET/CT; PSA: 65 days after study inclusion, + 67% compared to baseline)

Representative cases are provided in Figs. 3 and 4.

## Discussion

In the present prospective study, we evaluated the predictive performance of γ-H2AX and 53BP1 foci in PSMA-targeted RLT. There was a consistent trend emphasizing the predictive value of reduced DDR-markers at baseline for subsequent poor outcome under [^177^Lu]Lu-PSMA RLT, but likely due to the small patient cohort significance could not be reached. In this regard, low baseline DDR-marker levels prior to treatment on-set probably reflected low individual radiosensitivity and thus, were linked to early PSA-PD and shorter PSA-PFS. Thus, we herein provide for the first time data on the role of individual radiosensitivity in the context of PSMA-targeted RLT along with predictive capability, which requires further studies with larger patient populations to provide definite evidence. Nonetheless, the herein presented consistent results may also enable future studies investigating the potential of pretherapeutic administration of radiosensitizing agents, e.g., in patients having low baseline levels of γ-H2AX and 53BP1 foci.

Schumann et al. were the first to investigate changes in DDR-marker levels during [^177^Lu]Lu-PSMA RLT [27]. Enrolling 16 mCRPC patients, a distinctive time-dependent kinetic pattern of both γ-H2AX and 53BP1 foci was observed, with an early increase of both DDR-marker levels within + 4 h after administration and a subsequent decrease thereafter [27]. Ritt et al. corroborated these findings by reporting on similar DDR-marker kinetic patterns enrolling a larger cohort of 48 patients under ^177^Lu-DOTATOC (n = 26) or [^177^Lu]Lu-PSMA RLT (*n* = 22) [20]. Of note, in both studies, DDR-marker remained elevated compared to baseline even + 24 h after initiation of RLT [20, 27]. This is in line with our results also showing a time-dependent kinetic pattern of DDR-marker levels in a prospective cohort of 20 mCRPC patients. Of note, a recent study reported on the predictive value of DDR-markers for therapy response under ^177^Lu-DOTATATE [22], while the present investigation is the first to evaluate the predictive performance of γ-H2AX and 53BP1 foci for men treated with [^177^Lu]Lu-PSMA RLT. Consistent with findings of that study investigating DDR-markers in patients with gastroenteropancreatic neuroendocrine neoplasms treated with somatostatin receptor-targeted peptide receptor radionuclide therapy (PRRT), low levels of 53BP1 foci were linked to shorter PFS [22], supporting the notion of an incremental value of those DDR-markers for patients scheduled for endoradiotherapy.

Despite favorable results of PSMA-targeted RLT compared to other therapies in advanced disease [3, 9], a substantial portion of patients will experience early PD, and thus, reliable predictors for subsequent treatment response would be crucial for optimizing patient care [13]. In this regard, the averaged SUV_max_ derived from tumor lesions on pretherapeutic [^68^Ga]Ga-PSMA PET/CTs has shown promise for predicting early response to treatment and overall survival [11, 12]. However, due to various advantages such as improved image quality and longer half-life allowing for a higher throughput in a busy PET practice, recent years have witnessed an increased use of ^18^F-labeled PSMA radiotracers, e.g., [^18^F]F-PSMA-1007 [6]. Given this shift from ^68^Ga to ^18^F radiotracers in clinical routine, the predictive potential of ^18^F-PSMA PET in patients scheduled for RLT needs further exploration. We demonstrated that a low SUV_max_ derived from a pretherapeutic [^18^F]F-PSMA-1007 PET/CT was significantly associated with early PSA-PD after two cycles of RLT (*P* = 0.04). Nonetheless, SUV_max_ did not reach significance for the prediction of PSA-PFS in Kaplan–Meier analysis and univariate Cox-regression which might be clinically even more important than early PD for the long-term outcome of mCRPC patients. Although the study population was rather small, this may also be a hint at restricted predictive capability of ^18^F-PSMA PET-based quantification. While a strong relation between uptake of PSMA-ligands and histologic PSMA-expression has already been proven for [^68^Ga]Ga-PSMA-11 in a dedicated ex vivo animal model [28] and immunohistochemical validation of clinical primary prostate cancer samples [29], such evidence has not been provided for [^18^F]F-PSMA-1007 to date. However, there is an increasing body of evidence of potential pitfalls on scan interpretation for this ^18^F-labeled PSMA radiotracer [30]. Along with the high rate of indeterminate findings on ^18^F-PSMA especially in the skeleton [31] and moderate interobserver agreement rates [32], one may question the reliability of ^18^F-PSMA PET-based predictors. One way to maximize the predictive performance of PET-based parameters could be a dual tracer approach for patient selection targeting both PSMA and glucose metabolism [9]. Excluding patients with PSMA-negative and simultaneously FDG-positive metastases, Hofman et al. were able to achieve biochemical response in 65% of the RLT-treated patients compared to 46% in the VISION trial, in which only PSMA PET/CTs were performed for patient selection [3, 9].

In addition, blood-based markers such as AP have also shown promise for the prediction of treatment response [33, 34] and we also confirmed the value of AP levels in the present investigation (*P* = 0.﻿04), but inconsistent results question the suitability of this parameter to identify patients prone to early therapeutic failure [13]. For instance, Gafita et al. reported on no predictive value of AP for overall survival under [^177^Lu]Lu-PSMA RLT [35]. As such, liquid biopsies assessing DNA damage due to ionizing radiation, either emitted through external beam radiation or internal RLT, may provide a more causal-system approach to identify patients prone to treatment failure [17].

Following RLT-induced DSBs, a cascade of biological response mechanisms is activated including the phosphorylation of the histone H2AX to γ-H2AX and the assembling of 53BP1, subsequently leading to either repair, arrest, or apoptosis of the referring cell [15–17]. Thus, one might speculate that low levels of the DDR-markers 53BP1 and γ-H2AX are associated with reduced responsiveness to radiation subsequently causing ineffective cell repair and less apoptosis [36, 37]. In this regard Xiao et al. were able to demonstrate, that low levels of 53BP1 were associated with reduced expression of proteins fundamental for p53-dependent apoptosis such as the ataxia-telangiectasia mutated kinase and the checkpoint kinase-2 in a dedicated colorectal cancer cell line after radiation [38]. Decreased expression of 53BP1 led to radiotolerance with reduced apoptosis and elevated proliferation rates after treatment [38]. However, beta-irradiation due to ^177^Lu not only induces DDR mechanisms in malignant but also in benign cells, e.g., PBLs [20, 22]. Of note, the evolution of γ-H2AX and 53BP1 foci in PBLs have previously been investigated under RLT [20, 27].

In the present study, we expanded the concept of blood-based assessment of DNA damage and demonstrated clinical value of these parameters beyond dosimetry. We were able to demonstrate a clear and consistent trend emphasizing the predictive value of reduced DDR-markers assessed prior to treatment onset for subsequent poor outcome under [^177^Lu]Lu-PSMA RLT. Future studies, however, may also measure DDR-foci in circulating tumor cells (CTCs) possibly enabling an even more accurate determination of tumor radiosensitivity [21]. However, relative to PBLs, previous single-cell studies assessing mutations in CTCs of breast cancer patients revealed remarkable heterogeneity within investigated cells from the same patient [39], and thus, DDR-marker in CTCs derived from men affected with prostate cancer may also be prone to such intra-patient heterogeneity, limiting their usefulness for prediction.

The herein presented results also support the hypothesis that low DDR-marker levels reflect low individual radiosensitivity explaining their predictive value for treatment outcome. Although other clinical parameters demonstrated similar or even superior predictive performance, e.g., AP, this is of importance as these parameters can hardly be influenced. By contrast, simultaneous chemoradiotherapy to boost radiosensitivity upon external beam radiation is widely practiced in numerous other tumor entities e.g. non-small-cell lung cancer [40]. Thus, our study provides a rationale for future studies exploring the use of radiosensitizing agents under [^177^Lu]Lu-PSMA RLT, especially in high-risk individuals exhibiting low baseline DDR-marker levels. Among others, a combination of [^177^Lu]Lu-PSMA RLT and docetaxel has already demonstrated high response rates with only limited side effects [41]. An even more sophisticated approach was presented by Guo et al. [42]. Conjugating docetaxel-loaded nano-particles with a PSMA-ligand achieved high tumor doses while the whole-body dose was reduced [42]. Given the herein reported findings, patients with low γ-H2AX and 53BP1 foci in PBLs at baseline may benefit from such combination approaches, e.g., by conducting a docetaxel-based radiosensitizing prior to RLT.

This study has several limitations. First, although we were able to demonstrate consistent and robust results, the baseline DDR-marker closely failed to reach significance in predicting early PSA-PD and PSA-PFS due to the small number of enrolled patients in this prospective study. Therefore, future studies need to verify these results in a substantially larger cohort with a longer follow-up interval, which would then also allow to investigate overall survival. In addition, factors influencing the individual expression of DDR-markers in PBLs before RLT remain unclear and should also be investigated. Moreover, additional characterization of PBLs, e.g., CD4 + or CD8 + , might also help in better understanding radiosensitivity of specific immune cell types and their influence on treatment outcome after radiation therapy [43]. Future studies may also investigate the additional predictive role of DDR markers when combined with established outcome predictors, e.g., recently suggested nomograms [14].

## Conclusion

In this prospective study investigating mCRPC patients undergoing [^177^Lu]Lu-PSMA RLT, low baseline DDR-markers in PBLs demonstrated a trend towards association with poorer outcome. Although the study group was small and results need further confirmation to provide definite evidence, these preliminary findings lay the foundation for exploring additive radiosensitizing or treatment intensification in future studies with high-risk individuals scheduled for RLT.

## Supplementary Information

Below is the link to the electronic supplementary material.

ESM 1 (DOCX 102 KB)
