# Supplementary material for: Individual radiosensitivity reflected by γ-H2AX and 53BP1 foci predicts outcome in PSMA-targeted radioligand therapy
Source: Eur J Nucl Med Mol Imaging. 2022 Sep 22;50(2):602–12. doi: 10.1007/s00259-022-05974-8 (PMC9816192; doi:10.1007/s00259-022-05974-8)
Supplement: Supplementary file 1 — (DOCX 102 KB) [file 259_2022_5974_MOESM1_ESM.docx]

**Supplementary Material**

**Study protocol No. 9182_BO_S_2020**

Enrollment: 20 male patients between August 2020 and Mai 2021

Inclusion criteria:

- Written informed consent
- Affliction with mCRPC
  - Progressive disease under previous treatments including radiation therapy, androgen deprivation and second-line antihormonal treatment, and progress under or unfitness for chemotherapy
- [^18^F]F-PSMA-1007 PET/CT prior RLT with PSMA-avid disease

Exclusion criteria:

- Previous PSMA-targeted RLT
- According to EANM procedure guidelines (*Kratochwil* et al., EJNMMI, 2019)
  - Urinary tract obstruction or hydronephrosis on [^99m^Tc]Tc-MAG3 renal scintigraphy
  - Reduced renal function (eGFR <30ml/min)
  - Bone marrow insufficiency (leucocytes <2.5/μl; platelets < 75/nl)

Intervention:

- PSMA-targeted RLT with 7 to 7.5 GBq [^177^Lu]Lu-PSMA-I&T every 6-8 weeks until progression (PSA increase over 25% from nadir under RLT)

Examination:

- Collection of 3 blood samples (baseline, +1h and +24 after administration of [^177^Lu]Lu-PSMA-I&T) peritherapeutic around the first cycle [^177^Lu]Lu-PSMA RLT to determine DDR-marker expression through immunofluorescence microscopy

Follow-up:

- PSA measurements at and 4-6 weeks after each cycle of RLT until PSA-progression, termination of RLT due to other medical reasons or the end of follow-up period in December 2021

Endpoints:

- Early progressive disease following 2 cycles of RLT (PSA increase over 25% from baseline)
- PSA-progression-free survival

**Supplemental Table 1.** Baseline parameters and outcome of all patients

| ***Patient*** | ***Baseline*** | | | | | ***Outcome*** | | |
| --- | --- | --- | --- | --- | --- | --- | --- | --- |
|  | PSA (μg/l) | SUV_max_ | PSMA-TV (cm^3^) | γ-H2AX foci per cell | 53BP1 foci per cell | PSA (μg/l) after 2 cycles of RLT | PSA-change (%) after 2 cycles of RLT | PSA-PFS (months) |
| #1 | 38.9 | 13.84 | 23.96 | 0.48 | 0.5 | 25.3 | - 35 | 9 |
| #2 | 491 | 28.64 | 598.64 | 0,27 | 0.26 | 578 | + 18 | 3 |
| #3 | 336 | 13.21 | 139.57 | 0.38 | 0.36 | 1198 | + 257 | 2 |
| #4 | 108 | 16.14 | 87.1 | 0.53 | 0.53 | 172 | + 59 | 2 |
| #5 | 12 | 13.95 | 543.46 | 0.39 | 0.54 | 11.1 | - 8 | 3* |
| #6 | 136 | 24.65 | 434.54 | 0.37 | 0.51 | 4.3 | - 97 | 7 |
| #7 | 692 | 17.29 | 261.95 | 0.03 | 0.13 | 391.97 | - 43 | 2 |
| #8 | 15 | 4.697 | 16.33 | 0 | 0.02 | 35.8 | + 139 | 2 |
| #9 | 523 | 14.60 | 39.06 | 0.44 | 0.5 | 196 | - 63 | 6 |
| #10 | 768 | 9.46 | 546.17 | 0.13 | 0.27 | 1513 | + 97 | 1 |
| #11 | 85.1 | 9.98 | 284.27 | 0.10 | 0.33 | 329 | + 287 | 3 |
| #12 | 167 | 18.07 | 61.08 | 0.23 | 0.44 | 101 | - 40 | 4 |
| #13 | 59 | 18.39 | 130.15 | 0.16 | 0.18 | 98.7 | + 67 | 1 |
| #14 | 1812 | 21.57 | 539.4 | 0.13 | 0.24 | 1642 | - 9 | 5* |
| #15 | 48.8 | 8.78 | 80.17 | 0.1 | 0.19 | 57.6 | + 18 | 2 |
| #16 | 42.7 | 14.18 | 117.87 | 0.23 | 0.41 | 58.3 | + 37 | 1 |
| #17 | 242 | 49.9 | 157.55 | 0.69 | 0.54 | 235 | - 3 | 2* |
| #18 | 160 | 30.3 | 210.79 | 0.48 | 0.52 | 10.4 | - 94 | 6 |
| #19 | 234 | 44.05 | 134.65 | 0.14 | 0.32 | 75.2 | - 68 | 6* |
| #20 | 226 | 16.39 | 122.36 | 0.43 | 0.64 | 225 | 0 | 4 |
| *censored at last follow-up; PFS, progression-free survival; PSA, prostate-specific antigen; PSMA, prostate-specific membrane antigen; RLT, radioligand therapy; SUV, standardized uptake value; TV, tumor volume. | | | | | | | | |
